# A Novel Pre-treatment Model Predicting Risk of Developing Refractoriness to Transarterial Chemoembolization in Unresectable Hepatocellular Carcinoma

**DOI:** 10.7150/jca.44847

**Published:** 2020-05-18

**Authors:** Keshu Hu, Shenxin Lu, Miao Li, Feng Zhang, Bei Tang, Jia Yuan, Yan Shan, Pengju Xu, Rongxin Chen, Zhenggang Ren, Xin Yin

**Affiliations:** 1 Liver Cancer Institute & Zhongshan Hospital, Fudan University, Shanghai, China; Key Laboratory of Carcinogenesis and Cancer Invasion, Ministry of Education; 2 Department of Radiology, Zhongshan Hospital, Fudan University, Shanghai, China

**Keywords:** Hepatocellular carcinoma, Transarterial chemoembolization, Refractoriness, Risk stratification

## Abstract

**Background and aim**: Refractoriness to transarterial chemoembolization is common during the therapeutic process of hepatocellular carcinoma, which is an intractable issue and may compromise the prognosis. We aim to establish a pre-treatment model to identify patients with high risks of refractoriness.

**Methods**: From 2010 to 2016, 824 treatment-naive patients who had initially underwent at least two sessions of transarterial chemoembolization in Zhongshan Hospital, Fudan University were retrospectively enrolled. These patients were randomly allocated into a training cohort and a validation cohort. The pre-treatment scoring model was established based on the clinical and radiological variables using logistic regression and nomogram. The discrimination and calibration of the model were also evaluated.

**Results**: Logistic regression identified vascularization pattern, ALBI grade, serum alpha-fetoprotein level, serum γ-glutamyl transpeptidase level and major tumor size as the key parameters related to refractoriness. The p-TACE model was established using these variables (risk score range: 0-19.5). Patients were divided into six risk subgroups based on their scores (<4, ≥4, ≥7, ≥10, ≥13, ≥16). The discriminative ability, as determined by the area under receiver operating characteristic curve was 0.784 (95% confidence interval: 0.741-0.827) in the training cohort and 0.743 (95% confidence interval: 0.696-0.789) in the validation cohort. Moreover, satisfactory calibration was confirmed by Hosmer-Lemeshow test with P values of 0.767 and 0.913 in the training cohort and validation cohort.

**Conclusions**: This study presents a pre-treatment model to identify patients with high risks of refractoriness after transarterial chemoembolization and shed light on clinical decision making.

## Introduction

Hepatocellular carcinoma (HCC) is the sixth most common malignancy and the second leading cause of cancer-related death worldwide 1. According to a recent global view of HCC 2, almost 85% of HCC are estimated to occur in the developing area, particularly in Eastern Asia and sub-Saharan Africa. Besides, the HCC population in China, which are mainly ascribed to chronic hepatitis B virus (HBV) infection, are supposed to have an earlier onset of a mean age of 52 years old, and more than 60% of such patients present with intermediate-stage or advanced-stage HCC when diagnosed^2^. Transarterial chemoembolization (TACE) is currently the recommended treatment option for intermediate stage HCC with well-preserved liver function and performance status 3. TACE benefits patients in two ways: providing a treatment response and minimizing liver function damage by infusion embolization and chemotherapeutic agents into tumor feeding arteries 4. Nevertheless, in some cases, despite an initial response induced by TACE, repetitive TACE treatments could impair liver function gradually and induce stenosis of the hepatic artery. Even worse, TACE is associated with disturbances of tumor microenvironment, which results in hypoxia and upregulation of vascular endothelial growth factor (VEGF), thus promotes tumor invasion and metastasis 5. As such, with the increased sessions of TACE procedure, the treatment efficacy diminishes and patients then probably enter a state of so-called “TACE refractoriness”.

The concept of TACE refractoriness was first proposed by the Japan Society of Hepatology and updated as JSH-LCSGJ Criteria in 2014^4^, ^6^. According to these criteria, TACE-refractory HCC patients who belong to Child-Pugh grade A are candidates for molecular target therapy as a second-line treatment option 7. However, for patients with deteriorated liver function induced by repetitive TACE treatments, few subsequent treatment options are available and the prognosis is usually dismal 8, 9. If TACE refractoriness could be identified earlier, with compensated liver function, these patients could switch to molecular target therapy or other systematic treatments, avoiding exposure to the adverse effects of TACE. Actually, the necessity of early identification of TACE refractoriness in HCC population undergoing TACE treatments has been proposed by recent EASL guidelines 10.

Of note, the range of indications for TACE covers a wide spectrum in real-world clinical practice. Besides intermediate stage HCC, early stage HCC that deemed as ineligible for curative treatments due to significant liver impairment or general contraindication is also recommended as TACE candidates, according to the treatment stage migration principle 11. These patients are quite heterogeneous with different degrees of liver dysfunction and tumor burden, leading to a variable median overall survival of 13-43 months 12. Furthermore, treatment decisions in the individual patient are partially subjective, being largely dependent on the physician's decision within each institution. Development of an individual prediction model could allow for objective selection of ideal TACE candidates and guide decision for treatments. Indeed, there are several existing prognostic models such as hepatoma arterial-embolization prognostic (HAP) score 13, mHAP-II score^14^, the Assessment for Retreatment with TACE (ART) score^15^, etc. These existing prognostic models, however, have been questioned due to being incapable to predict patient outcome accurately in independent validation cohorts, causing doubt upon their clinical utility^16^, ^17^. Till now, no pre-treatment model evaluating the risk of TACE refractoriness has been established. Herein, we developed a novel scoring model based on pre-treatment clinical and radiological parameters for the early identification of TACE-refractoriness in early/intermediate stage HCC following TACE treatment.

## Methods

### Patients

From 2010 to 2016, patients who were diagnosed with unresectable HCC and received TACE treatment at liver cancer institute, Zhongshan hospital were screened. The unresectable HCC was defined according to the comprehensive assessment of the size, number, location of the tumors as well as the preserved liver function of the patient. Patients were included who met the following criteria: 1) with HCC diagnosis confirmed by pathological or clinical diagnosis according to the AASLD criteria 18; 2) with BCLC stage A/B stage HCC that had undergone at least two sessions of TACE treatment; 3) with Child-Pugh grade of A or B before treatment; 4) with Eastern Cooperative Oncology Group performance status score of 0-1. Patients were excluded if they: 1) had received other anti-tumor treatments prior to TACE; 2) with serious dysfunction of the heart or kidney; 3) with other malignancies in addition to HCC. Patients were 1:1 assigned to training cohort and validation cohort by the random numbers generated by the software Stata. This study was approved by the institutional review board of Zhongshan Hospital and complied with the standards of the Declaration of Helsinki and current ethical guidelines.

### TACE procedure, follow up and evaluation of TACE refractoriness

TACE was performed by the same team of hepatologists with more than 10-year experience, under the institutional standard protocol as described by Yin et al. 19. Briefly, a 5F or 4F catheter was introduced into the abdominal aorta under fluoroscopy guidance. Angiography was performed to identify the tumors and their feeding hepatic arteries. Anticancer drugs (1000 mg of 5-fluorouracil, 100~150 mg of oxaliplatin) were infused into the tumor feeding arteries. Super-selective embolization was performed by using a microcatheter if needed. An emulsion of 5 to 20 mL lipiodol with 30 mg epirubicin was slowly injected into the feeding arteries.

Four weeks after treatment, patients were followed by computed tomography (CT) and/or magnetic resonance imaging (MRI), liver function, blood routine test and tumor markers. The effects of TACE were assessed by using dynamic CT/MRI and residual enhancement of nodules was measured with consideration of the modified response evaluation criteria in solid tumors (mRECIST). If no residual viable tumors were indicated based on imaging results, patients were followed up every 2 or 3 months for one year and every six months thereafter. If residual viable or newly developed tumors were identified, repeated TACE were performed on an “on-demand” basis depending on individual tumor response and hepatic functional reserve 20.

TACE refractoriness was judged by the our hepatologists according to JSH-LCSGJ Criteria: two or more consecutive insufficient responses of the treated tumor (viable lesion >50%) or increases of tumor number after changing the chemotherapeutic agents and/or reanalysis of the feeding artery seen on response evaluation CT/MRI at 1-3 months after having adequately performed selective TACE; the appearance of vascular invasion; the appearance of extrahepatic spread; a continuous elevation of tumor markers immediately after TACE even though slight transient decrease is observed^4^.

### Clinical and radiological variables contributing to develop prediction model

To evaluate the potential predicting variables, the clinical variables included the patient's age, sex, etiology, laboratory tests, tumor biomarkers, tumor size, tumor number and radiological features. The ALBI score was calculated and graded as indicated by previous studies 21. A preoperative neutrophil-to-lymphocyte ratio (NLR) was also calculated within 7 days before TACE. Additionally, the radiological features, especially the enhancement characteristics of the main target tumors were determined based on four-fold categorization of HCC vascularization patterns 22, 23 on baseline CT/MRI. Two experienced radiologists who did not know baseline clinical data assessed overall tumor response and vascularization patterns. In detail, Type-1 represented a “homogeneous enhancement” pattern with no increase in arterial blood flow. The Type-2 pattern represented “homogeneous enhancement” pattern with increased arterial blood flow. Type-3 is a heterogeneous enhancement pattern with a septum-like structure; and Type-4 is an irregularly shaped ring structure enhancement pattern. Typical images of vascularization pattern were illustrated in Figure S1. These clinical and laboratory variables were measured before TACE therapy and were collected from patients' records.

### Statistical analysis

This analysis was reported according to the TRIPOD (Transparent reporting of a multivariable prediction model for individual prognosis or diagnosis) guidelines 24. Continuous variables were presented as an average ± standard deviation or as the median and its range. The normal distribution of the continuous variables were tested by Shapiro-Wilk tests, and the subsequent hypothesis tests were performed by either student t-test for normal distributed variables, or rank-sum (Mann-Whitney) test for abnormal distributed variables. Categorical variables were tested by Fisher exact test. The risk factors related to TACE refractoriness were evaluated by logistic regression, and all the variables except for albumin, bilirubin and Child-Pugh grade were included in the multivariate regression, in case of the collinearity. The cut-off levels of the specific factors were determined by receiver operating characteristic curve (ROC). The development of the pre-treatment scoring model was also based on the logistic regression of multiple variables, which was illustrated as a nomogram as well. The discrimination efficacy of the model was examined by the area under ROC (AUROC), and the goodness of fit were validated by calibration curve and Hosmer-Lemeshow (H-L) test, in which case P value > 0.05 indicated good performance. All the statistical analyses were conducted by software Stata 14.0 for Windows (StataCorp, College Station, TX) or R language (version 3.5.2; R Package for Statistical Computing; www.r-project.org). P < 0.05 was considered statistically significant.

## Results

### Clinicopathological characteristics

From January 2010 to December 2016, a total of 1661 patients were screened and 824 eligible patients were enrolled and randomly assigned to training cohort (n=412) and validation cohort (n=412). Patient characteristics are summarized in Table 1. The clinicopathological characteristics of the patients does not differ significantly between the training cohort and validation cohort (All P > 0.05). Generally, most of the patients were male (n=698, 84.7%) and with HBV infection (n=654, 79.4%). The patients enrolled in the present study had tumors with the median major size of 6.0cm (range: 0.5-21.5cm) and the median number of 1 (range: 1-9), of whom 57.2% (n=471) patients had a single tumor. The majority of the patients were diagnosed as BCLC stage A (n=502, 60.9%) with a favorable liver function of Child-Pugh grade A (n=804, 97.6%). The median TACE times that all the patients had undergone were 3 times (range: 2-16), and approximately half (n=406, 49.3%) of the patients eventually developed TACE refractoriness judged by JSH-LCSGJ Criteria 4.

### Risk factors and cut-offs

All the potential risk factors were summarized in Table 2 and univariate logistic regression was performed in order to calculate the unadjusted odds ratio (OR). Except for the ALBI grade, the other associated factors including serum total bilirubin, serum albumin and Child-Pugh grade were excluded from the further multivariate regression ascribed to the collinearity. ALBI grade was retained referring to the previous study 25. As a result, the vascularization pattern, ALBI grade and major tumor size demonstrated obvious significance in TACE refractoriness (Table 2). Considering that the serum alpha-fetoprotein (AFP) level and γ-glutamyl transpeptidase (γ-GT) played an important role in clinical practice, and statistical differences were observed in univariate regression, eventually they were employed for the establishment of the model as well, together with the vascularization pattern, ALBI grade and major tumor size.

AUROC was calculated to determine the optimal cut-off values of AFP, γ-GT and the major tumor size, to establish an easy-to-use prediction model and simultaneously maintain its discrimination efficacy. The ideal single cut-off level of the three values calculated by Youden index were 78ng/ml (Youden index=0.224), 69.5U/L (Youden index=0.218) and 6.1cm (Youden index=0.198). Afterwards, slight adjustments were made to maintain the accuracy and for a convenient usage. Respectively, the cut-offs were 80ng/mL, 4000ng/mL for AFP; 50U/L, 75U/L, 135U/L for γ-GT; and 5cm for major tumor size (Table S1). The Youden indexes were 0.217 (80ng/mL), 0.153 (4000ng/mL), 0.159 (50U/L), 0.210 (75U/L), 0.175 (135U/L), and 0.184 (5cm) for the referred parameters, respectively. The ROC curves were shown in Figure S2.

### Development of the p-TACE model

**Figure 1:**
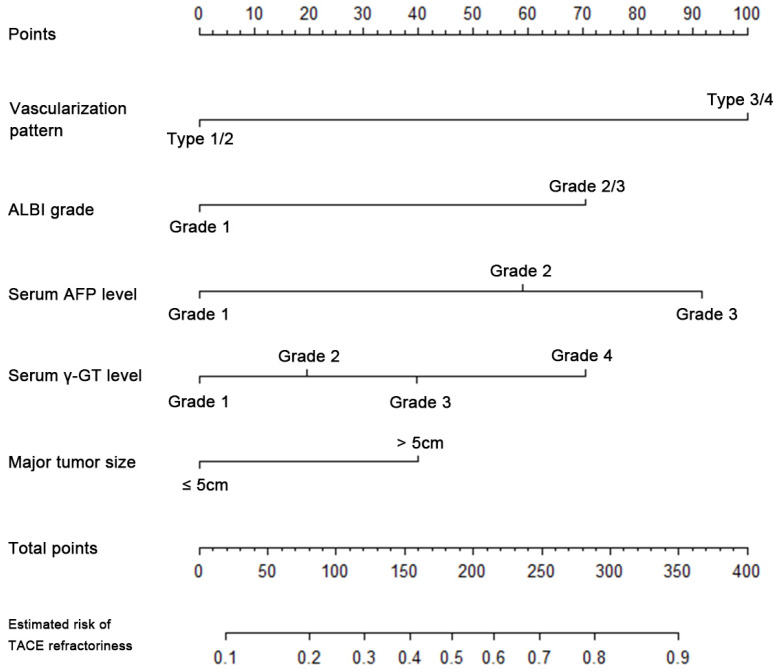
The nomogram of logistic regression model for TACE refractoriness in training cohort. TACE, transarterial chemoembolization; ALBI grade, albumin-bilirubin grade; AFP, alpha-fetoprotein; γ-GT, γ-glutamyl transpeptidase

Logistic regression of the risk factors for TACE refractoriness, including vascularization pattern, ALBI grade, AFP grade, γ-GT grade and major tumor size, was performed in the training cohort (n=412). The results were summarized in Table 3 and a nomogram was illustrated in Figure 1. For a convenient but accurate prediction, the scoring model was further developed on the basis of the logistic regression and nomogram. Accordingly, the model sum the respective score of five factors, as described in Table 4. Furthermore, the prediction efficacy was evaluated by the comparison between the p-TACE model vs. the logistic regression model in AUROC (0.784 vs. 0.787) and H-L test (P=0.767 vs. P=0.748), suggesting a satisfactory maintenance of prediction efficacy in the new scoring model.

### Validation of the p-TACE model

**Figure 2:**
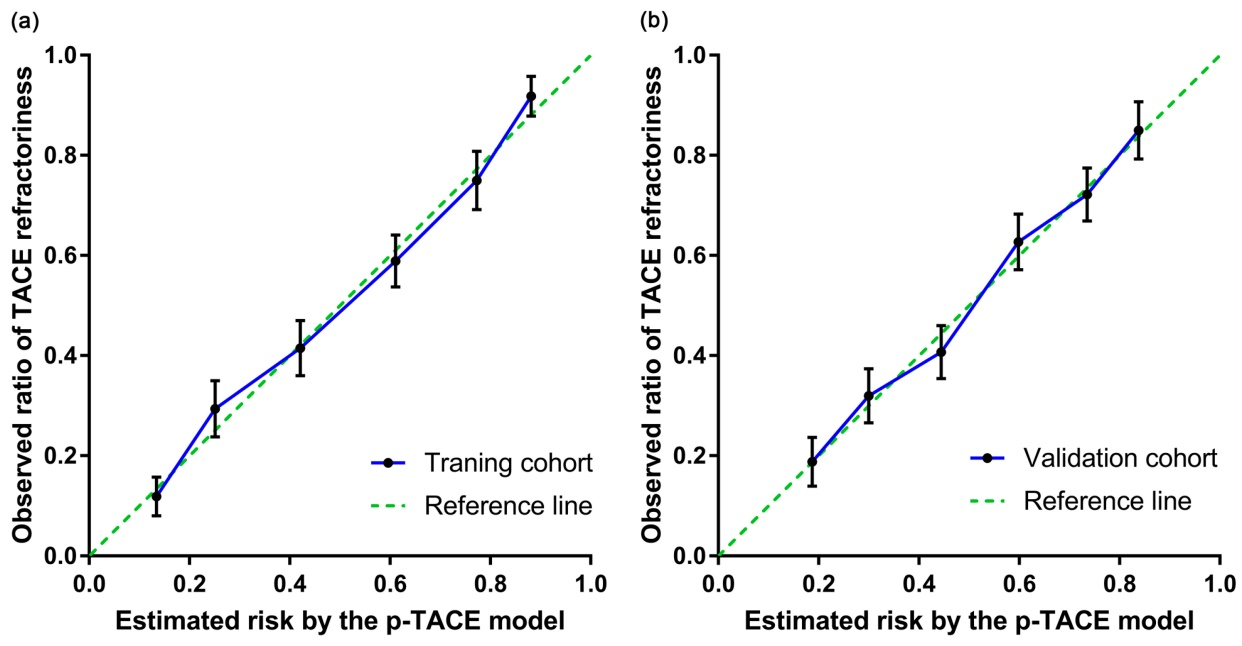
The calibration curve of the p-TACE model in training and validation cohort. (a) the calibration curve of training cohort; (b) the calibration cure of validation cohort. TACE, transarterial chemoembolization

The prediction efficacy of p-TACE scoring model was further evaluated in the validation cohort (n=412) as well. Consistent results were obtained in both training cohort and validation cohort, including AUROC (0.784 [95% CI: 0.741-0.827] vs. 0.743 [95% CI: 0.696-0.789]), H-L test (P=0.767 vs. 0.913), risk stratification (Table 5) and calibration curves (Figure 2). All these results confirmed the favorable predictive capacity of the novel model in identifying the risk of TACE-refractoriness prior to TACE treatment.

## Discussion

In present study, we established a pre-treatment scoring model (p-TACE model) based on five common clinical and radiological variables. The p-TACE model successfully stratified patients according to their risks of developing TACE refractoriness. With good discrimination efficacy and calibration, this easy-to-use model facilitates the pre-treatment individualized prediction of TACE refractoriness and contributes to clinical decision making.

Our model was developed based on the vascularization pattern, ALBI grade, AFP level, γ-GT level and major tumor size, which were significant factors associated with TACE refractoriness in logistic regression model. To be specific, the vascularization pattern and the major tumor size or tumor burden affects the treatment efficacy of TACE; ALBI grade is an assessment of liver function reserve and associated with HCC prognosis 26; AFP level is related to the differentiation of the cancer 27; and γ-GT level has been reported to be associated with tumor relapse and poor survival outcome 28. These synergistic effects provided a rationale for the prediction efficacy of the novel p-TACE scoring model.

Compared to the nomogram, the p-TACE scoring model is more convenient for clinical usage, with an equal prediction efficacy proved by AUROC and H-L test. According to our model, patients with a high risk of developing TACE refractory status (eg, >50%) should not receive repetitive TACE. Treatment migration strategy such as molecular target therapy, immunotherapy should be considered, since they were probably TACE non-responders and would not benefit from TACE monotherapy. In accordance, the OPTIMIS study revealed that continuing TACE after TACE refractoriness would lead to deterioration of liver function and unfavorable prognosis 29. More recently, the results from a randomized, multicenter, phase II clinical trial (TACTICS trial) indicated that TACE plus Sorafenib significantly improved progression-free survival over TACE alone in patients with intermediate HCC 30. The duration time of Sorafenib administration was positively associated with progression-free survival outcome. Thus, early introduction of effective systematic treatment to patients with high risk of TACE refractoriness could improve prognosis in comparison with TACE monotherapy. This treatment migration concept is supported by the ESMO clinical practice guideline for HCC 31.

The p-TACE model is established to predict the risk of TACE refractoriness prior to TACE treatment. In the model, we employed an important factor, the homogeneous/heterogeneous vascularization of the tumor. Several lines of studies indicated the post-treatment lipiodol deposition and the response to TACE were correlated with tumor vascularization 32, 33. Besides, it has been reported that heterogeneous vascularization within the tumor is associated with poorly differentiated HCC cells 34. After repetitive TACE treatment, these poorly differentiated tumor cells are probably empowered with stemness properties and chemoresistance under hypoxia microenvironment induced by TACE 35, 36. Hence, with the introduction of this important radiological feature, the p-TACE model was able to predict TACE refractoriness risk accurately before on-demand TACE. The underlying mechanism of the correlations among heterogeneous vascularization, poor tumor differentiation and refractoriness to TACE needs further exploration.

There are some limitations in the present study. Firstly, given that the majority of patients included in this study were patients with HBV-related HCC, the prediction efficacy of p-TACE model might not be consistent for Western patients with HCV-related HCC. Secondly, although the p-TACE scoring model was derived and internally validated on a larger cohort from an Asian liver cancer center, external validation in different regions is necessary. Thirdly, although molecular target therapy may be potentially effective for HCC patients with TACE refractory status, the optimal treatment modality has not been determined in present study. However, the strength of our study lies in: 1) employing accessible clinical parameters and developing an easy-to-use model for clinical application; 2) including a radiological factor of vascularization and assessing tumor features in a visualized manner; 3) identifying pre-treatment TACE refractoriness risk and facilitating early treatment migration. To the best of our knowledge, the p-TACE model is the first pre-treatment model for predicting TACE refractoriness risk in early/intermediate stage HCC.

In conclusion, we successfully derived and validated a pre-treatment scoring model, exhibiting adequate performance for individual prediction of TACE refractoriness in early/intermediate-stage HCC patients following TACE treatment. Further validation of the novel scoring model based on prospective cohorts are warranted.

## Supplementary Material

Supplementary figures and tables.

## Figures and Tables

**Table 1:** The baseline characteristics of the patients in training cohort (n=412) and validation cohort (n=412)

	Total (n=824)	Training Cohort (n=412)	Validation Cohort (n=412)	
Gender, (male/female)	698/126	345/67	353/59	P=0.498
Age, years	59.2±11.8	59.2±11.7	59.3±11.9	P=0.920
HBV infection, (no/yes)	170/654	79/333	91/321	P=0.344
BCLC stage, (A/B)	502/322	245/167	257/155	P=0.432
Serum total bilirubin, μmol/L	13.9±7.8	13.7±7.7	14.1±7.8	P=0.331
Serum γ-GT, U/L	95 [12 ~ 998]	94 [12 ~ 998]	98 [16 ~ 827]	P=0.356
Serum albumin^†^, g/L	38.3±5.3	38.1±5.2	38.4±5.5	P=0.399
Serum AFP, ng/ml (median[range])	107[1 ~ >60500]	125.5[1 ~ >60500]	99.5[1 ~ >60500]	P=0.219
Child-Pugh grade, (A/B)	804/20	400/12	404/8	P=0.498
Neutrophil-to-lymphocyte ratio, (median[range])	3[0.5 ~ 86.7]	2.9[0.7 ~ 53.1]	3.1[0.5 ~ 86.7]	P=0.133
Major tumor size group, (≤5cm/>5cm)	355/469	186/226	169/243	P=0.260
Number of tumors, (single/multiple)	471/353	227/185	244/168	P=0.260
Vascularization patterns, (type 1&2 / type 3&4)	455/369	222/190	233/179	P=0.484
ALBI grade^†^, (1/2/3)	399/415/9	195/212/5	204/203/4	P=0.778
TACE times, (median[range])	3[2~16]	3[2~13]	4[2~16]	P=0.233
TACE refractoriness, (no/yes)	418/406	210/202	208/204	P=0.944

^†^1 value for albumin and ALBI grade were missing HBV, hepatitis B virus; BCLC stage, Barcelona Clinic Liver Cancer stage; γ-GT, γ-glutamyl transpeptidase; AFP: alpha-fetoprotein; ALBI grade: albumin-bilirubin grade; TACE: transarterial chemoembolization

**Table 2:** The respective OR of the possible risk factors related to TACE refractoriness

Risk factors	Unadjusted OR [95% CI]	P value	Adjusted OR [95% CI]^‡^	P value
Gender, (male/female)	1.306[0.891-1.913]	0.171	1.451[0.872-2.416]	0.152
Age, years	0.993[0.982-1.005]	0.250	0.997[0.980-1.015]	0.765
HBV infection, (no/yes)	1.421[1.011-1.997]	0.043^*^	1.553[0.938-2.574]	0.087
BCLC stage, (A/B)	1.049[0.793-1.388]	0.738	1.060[0.595-1.888]	0.843
Serum total bilirubin, μmol/L	1.021[1.003-1.040]	0.021^*^	NA	-
Serum γ-GT, U/L	1.003[1.002-1.004]	<0.001^*^	1.001[0.999-1.002]	0.363
Serum albumin, g/L	0.429[0.322-0.572]	<0.001^*^	NA	-
Serum AFP, ng/ml	1.000[1.000-1.000]^†^	<0.001^*^	1.000[1.000-1.000]^†^	0.089
Child-Pugh grade, (A/B)	0.839[0.344-2.046]	0.699	NA	-
Neutrophil-to-lymphocyte ratio	1.007[0.975-1.040]	0.671	1.015[0.980-1.051]	0.412
Major tumor size, cm	1.124[1.078-1.172]	<0.001^*^	1.106[1.035-1.182]	0.003^*^
Number of tumors	0.998[0.913-1.092]	0.968	1.003[0.838-1.201]	0.971
Vascularization pattern, (type 1&2 / type 3&4)	3.769[2.820-5.038]	<0.001^*^	3.473[2.354-5.124]	<0.001^*^
ALBI grade, (1/ 2&3)	2.608[1.983-3.431]	<0.001^*^	2.379[1.648-3.434]	<0.001^*^

^†^The unadjusted OR of serum AFP level should be 1.00003 (95% CI: 1.00002 ~ 1.00004), and the adjusted be 1.00001 (95% CI: 0.999998-1.000028) to be exact. ^‡^The parameters serum total bilirubin, serum albumin and Child-Pugh grade were excluded from the multivariate logistic regression due to the collinearity with ALBI grade. OR, odds ratio; CI, confidence interval; HBV, hepatitis B virus; BCLC stage, Barcelona Clinic Liver Cancer stage; γ-GT, γ-glutamyl transpeptidase; AFP: alpha-fetoprotein; ALBI grade: albumin-bilirubin grade; NA, not applicable ^*^Statistically significant (P<0.05)

**Table 3:** The results of logistic regression in training cohort (n=412)

Risk factors	Coefficient	OR[95% CI]	P value
Vascularization pattern (Type 3/4 vs. Type 1/2)	1.327	3.769[2.403-5.911]	<0.001^*^
ALBI grade (Grade 2/3 vs. Grade 1)	0.936	2.551[1.625-4.004]	<0.001^*^
Major tumor size (>5cm vs. ≤5cm)	0.531	1.700[1.050-2.752]	0.031^*^
AFP grade			
Grade 2 vs. Grade 1	0.783	2.188[1.331-3.597]	0.002^*^
Grade 3 vs. Grade 1	1.217	3.377[1.779-6.413]	<0.001^*^
γ-GT grade			
Grade 2 vs. Grade 1	0.259	1.296[0.629-2.671]	0.482
Grade 3 vs. Grade 1	0.526	1.692[0.873-3.277]	0.119
Grade 4 vs. Grade 1	0.935	2.547[1.310-4.952]	0.006^*^
Constant	-5.242	-	-

OR: odds ratio; ALBI grade: albumin-bilirubin grade; AFP: alpha-fetoprotein; γ-GT, γ- glutamyl transpeptidase ^*^Statistically significant (P<0.05)

**Table 4:** The novel scoring model (p-TACE model) for pre-treatment prediction of TACE refractoriness

Risk factors	Grade	Score			
**Vascularization pattern**	Type 1/2	0			
	Type 3/4	5.5			
**ALBI grade**	1	0			
	2/3	3.5			
**AFP level, ng/mL**	~80	0			
	~4000	3			
	>4000	5		**Scoring model grade (score)**	**Estimated risk of TACE refractoriness**
**γ-GT level, U/L**	~50	0			
	~75	1		Grade 1 (<4)	15% or less
	~135	2		Grade 2 (≥4)	appr. 30%
	>135	3.5		Grade 3 (≥7)	appr. 40%
**Major tumor size, cm**	~5	0		Grade 4 (≥10)	appr. 60%
	>5	2		Grade 5 (≥13)	appr. 75%
**Total**		range 0 ~ 19.5		Grade 6 (≥16)	85% or more

TACE: transarterial chemoembolization; ALBI grade: albumin-bilirubin grade; AFP: alpha-fetoprotein; γ-GT, γ- glutamyl transpeptidase

**Table 5:** The estimated and observed risks of TACE-refractoriness by the p-TACE model in training and validation cohort

Scoring model	Training cohort (n=412)		Validation cohort (n=412)	
	Estimated risk (%)	Observed ratio [95% CI] (%)	Estimated risk (%)	Observed ratio [95% CI] (%)
Grade 1	13.4	11.9[6.0 - 22.2]	18.7	18.8[10.9 - 30.3]
Grade 2	25.1	29.4[19.8 - 41.4]	30.0	32.0[22.4 - 43.4]
Grade 3	42.1	41.5[31.3 - 52.4]	44.4	40.7[30.8 - 51.4]
Grade 4	61.1	58.9[48.4 - 68.6]	59.8	62.7[51.2 - 72.9]
Grade 5	77.3	75.0[61.9 - 84.7]	73.5	72.2[60.7 - 81.4]
Grade 6	88.1	91.8[80.0 - 96.9]	83.8	85.0[70.1 - 93.2]

CI: confidence interval

## Abbreviations

HCC: Hepatocellular carcinoma
HBV: hepatitis B virus
TACE: transcatheter arterial chemoembolization
VEGF: vascular endothelial growth factor
HAP: hepatoma arterial-embolization prognostic
ART: Assessment for Retreatment with TACE
CT: computed tomography
MRI: magnetic resonance imaging
mRECIST: modified response evaluation criteria in solid tumors
NLR: neutrophil-to-lymphocyte ratio
TRIPOD: transparent reporting of a multivariable prediction model for individual prognosis or diagnosis
ROC: receiver operating characteristic curve
AUROC: area under receiver operating characteristic curve
H-L test: Hosmer-Lemeshow test
OR: odds ratio
AFP: alpha-fetoprotein
γ-GT: γ-glutamyl transpeptidase
CI: confidence interval
