# Supplementary material for: A Novel Pre-treatment Model Predicting Risk of Developing Refractoriness to Transarterial Chemoembolization in Unresectable Hepatocellular Carcinoma
Source: J Cancer. 2020 May 18;11(15):4589–96. doi: 10.7150/jca.44847 (PMC7255373; doi:10.7150/jca.44847)
Supplement: Supplementary file 1 — Supplementary figures and tables. [file jcav11p4589s1.pdf]

Table S1. The cut-off level of AFP, γ-GT and major tumor size

	Grade 1	Grade 2	Grade 3	Grade 4	AUROC	Original AUROC
AFP	~80ng/mL	~4000ng/mL	>4000ng/mL	-	0.630	0.643
γ-GT	~50U/L	~75U/L	~135U/L	>135U/L	0.633	0.629
major tumor size	~5cm	>5cm	-	-	0.592	0.616

AUROC, area under receiver operating characteristic curve; AFP: alpha-fetoprotein; γ-GT, γ-glutamyl transpeptidase

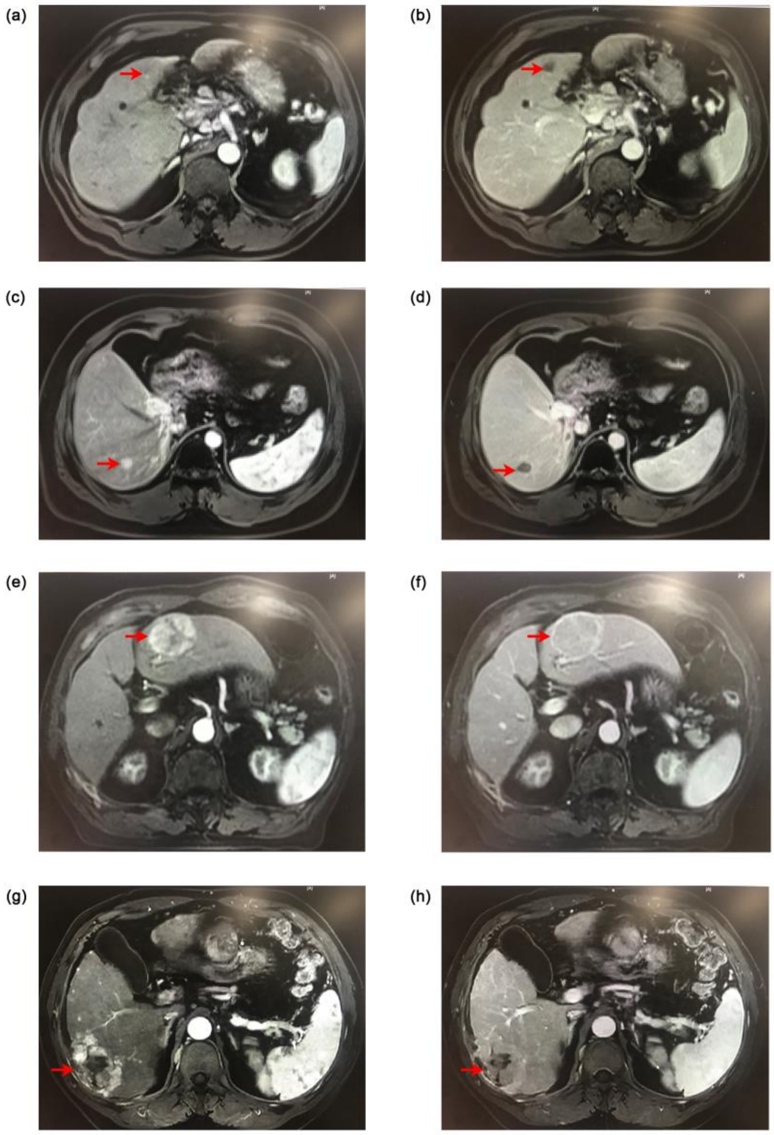

Figure S1. The typical images of four different vascularization patterns of HCC by enhanced magnetic resonance

(a) vascularization pattern type-1 in hepatic arterial phase; (b) vascularization pattern type-1 in portal vein phase; (c) vascularization pattern type-2 in arterial hepatic phase; (d) vascularization pattern type-2 in portal vein phase; (e) vascularization pattern type-3 in arterial hepatic phase; (f) vascularization pattern type-3 in portal vein phase; (g) vascularization pattern type-4 in arterial hepatic phase; (h) vascularization pattern type-4 in portal vein phase

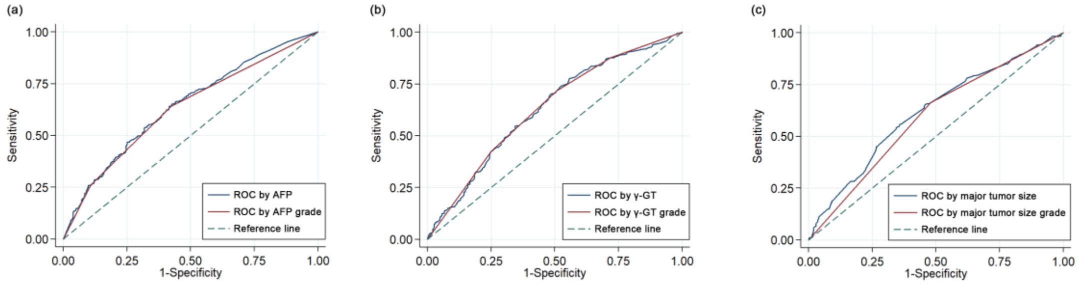

Figure S2. ROC curves of cut-off levels for risk factors AFP, γ-GT and major tumor size

(a) ROC by AFP; (b) ROC by γ-GT; (c) ROC by major tumor size

ROC, receiver operating characteristic curve; AFP, alpha-fetoprotein; γ-GT, γ-glutamyl transpeptidase
